# *Ranunculus Yuexiensis* (Ranunculaceae): A New Species From Dabie Mountains, China, Based on Plastome and Morphological Data

**DOI:** 10.1002/ece3.72418

**Published:** 2025-10-28

**Authors:** Lang Shen, Hai‐Jun Ma, Wei‐Qi Meng, Lu‐Jing Wang, Rong‐Bin Wang, Xiang Wang, Wan‐Zeng Wang, Guo‐Sheng Lv, Jian‐Wen Shao, Kun Liu

**Affiliations:** ^1^ Anhui Provincial Key Laboratory of the Conservation and Exploitation of Biological Resources, College of Life Sciences Anhui Normal University Wuhu Anhui China; ^2^ Institute of Botany, Jiangsu Province and Chinese Academy of Sciences (Nanjing Botanical Garden Mem. Sun Yat‐Sen) Nanjing China; ^3^ Innovative Research Team for Forest Restoration Mechanisms, Chishui National Ecological Quality Comprehensive Monitoring Stations, Nanjing, Institute of Environmental Sciences, Ministry of Ecology and Environment of the People's Republic of China (MEE) Nanjing China; ^4^ Institute of Chinese Medicine Resources Anhui College of Traditional Chinese Medicine Wuhu Anhui China

**Keywords:** China, new species, phylogeny, plastome, r*anunculus*

## Abstract

A new species of Ranunculaceae, *Ranunculus yuexiensis*, is described and illustrated from the Dabie mountains, China. *R*. *yuexiensis* is similar to *R*. *ternatus* in having root tubers. However, it can be readily distinguished from *R*. *ternatus* by its simple and fewer basal leaves, larger flower size, long petioles on the lower stem leaves, and puberulent (vs. glabrous) carpels and achenes. The complete plastomes of this new species are 155,537–156,009 bp in length and exhibit a typical quadripartite structure. Phylogenetic analysis, based on plastome data, shows that *R*. *yuexiensis* is closely related to *R*. *ternatus*, which is consistent with our morphological analysis.

## Graphical abstract

A new species, Ranunculus yuexiensis L. Shen, H. J. Ma & Kun Liu sp. nov. from Dabie mountains, China. R. yuexiensis is similar to R. ternatus in having root tubers. However, it can be readily distinguished from R. ternatus by its simple and fewer basal leaves, larger flower size, long petioles on the lower stem leaves, and puberulent carpels and achenes.

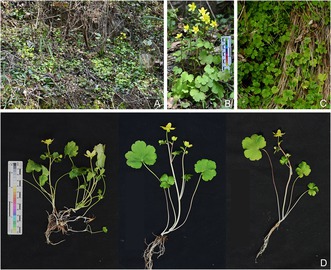

## 1 Introduction

*Ranunculus* L., consisting of about 600 species, is the largest genus in the Ranunculaceae and is widely distributed in the world (Hörandl and Emadzade 2012; Fei et al. 2023c). Over the past 10 years, some new species in *Ranunculus* are still being found (W. T. Wang 2015, 2016, 2018, 2019a, 2019b; J. Wang 2022; W. T. Wang 2022; Wang and Chen 2015; Yuan and Yang 2017a, 2017b, 2017c; Erkul et al. 2021; Shchegoleva et al. 2022; Fei et al. 2022, 2023a, 2023b; Dunkel 2024). Meanwhile, some species have been confirmed as synonymous, such as *R*. *kangmaensis* (Zhang et al. 2020), *R*. *huainingensis*, and *R*. *lujiangensis* (Fei et al. 2022). In China, one of the greatest concentrations of *Ranunculus* species, more than 150 species and 30 varieties are currently recognized in the genus (Wang and Gilbert 2001; Fei et al. 2022, 2023a, 2023b). The Dabie Mountains are located at the junction of Anhui, Hubei, and Henan provinces, and only 7 species of *Ranunculus* have been recorded in this region (J. Wang 2022; W. T. Wang 2022), namely *Ranunculus cantoniensis*, *R. chinensis* , *R. japonicus* , *R. muricatus* , *R. sceleratus* , *R. sieboldii* , and *R. ternatus*. In traditional Chinese medicine (TCM), the heat‐clearing and detoxifying properties of this genus are highly regarded. Nevertheless, certain fresh specimens of these plants contain mild toxicity, which can be neutralized through either drying or heating processes (Dai et al. 2024).

During a botanical expedition to the Dabie Mountains, a key biodiversity hotspot in China, conducted from March to April 2024, we discovered an unusual population of *Ranunculus* at the Tianxia scenic area in Yuexi county, located in southwestern Anhui Province, China. Subsequently, we discovered some larger populations of this unidentified species in neighboring Yingshan County, Hubei Province. The plants are somewhat similar to *R. ternatus* Thunberg, a species also occurring in Anhui and Hubei Province, in having root tubers, but differ by an array of characters, such as the size and shape of basal and cauline leaves, size of flowers, size and shape of petals, and puberulous receptacles, carpels, and achenes.

## 2 Materials and Methods

### 2.1 Morphological Comparison

Specimens and individual plants of *Ranunculus yuexiensis* were collected from wild populations in Yuexi County, Anhui Province, and Yingshan County, Hubei Province, China, for morphological comparison. The populations of this putative new taxon in both localities are extensive, comprising thousands of individuals. Some individuals were also transplanted and cultivated in the Botanical Garden of Anhui Normal University, Wuhu, for chloroplast genome analysis and further observation.

Gross morphology and phenological data were recorded during field expeditions. Descriptions were based on observations of living plants in their natural habitat as well as those cultivated in the Botanical Garden. No morphological variation was observed in the new species when compared across cultivated and wild habitats.

### 2.2 Phylogenetic Reconstruction

Three representative individuals of the putative new species across different populations were selected for further molecular analysis: one from the type population in Yuexi County and two from Yingshan County. Based on morphological similarity, two species (*R*. *ternatus* and *R*. *polii*) were additionally included for plastome sequencing. Voucher specimens were deposited in the herbarium of Anhui Normal University (AHNB) (Table 1). Fresh leaves of these samples were collected and shipped with ice packs to a sequencing company for next‐generation sequencing (NGS). Approximately 4 GB of raw data for each sample was obtained. Plastome assembly was conducted using the widely used software GetOrganelle (Jin et al. 2020). The assembly results were annotated using the online tool GeSeq (https://chlorobox.mpimp‐golm.mpg.de/geseq.html) (annotated reference genome: *R*. *ternatus* NC 081908). Additionally, the online website Chloroplot (https://irscope.shinyapps.io/Chloroplot/) was utilized to map the chloroplast genomesphere.

**TABLE 1:** Origins and vouchers of six newly sequenced *Ranunculus*.

Order	Code name	Species	Localities (voucher)	Coordinates	Altitude (m)	Voucher specimen	GenBank
1	RA1	*R*. *yuexiensis*	Tianxia scenic spot, Hetu Town, Yuexi, Anhui	30.817844° N/116.064770° E	428	LK240401	PV573921
2	RA2	*R*. *yuexiensis*	Wenquan Town, Yingshan, Hubei	30.70371138° N/115.74500325° E	384	LK250308	PV573920
3	RA3	*R*. *yuexiensis*	Qizhangya scenic spot, Nanhe Town, Yingshan, Hubei	30.55384538° N/115.67044938° E	275	LK250202	PV546306
4	RA4	*R. ternatus*	Zheshan, Wuhu, Anhui	31.33975303° N/118.37770013° E	68	SL250301	PV579837
5	RA5	*R. ternatus*	Nanjing Botanical Garden Mem. Sun Yat‐Sen, Nanjing, Jiangsu	32.05883041° N/118.83265624° E	58	MHJ25022701	PV579838
6	RA6	*R. polii*	Fanglan Lake Wetland, Jiujiang, Jiangxi	29.66571167° N/116.07637489° E	15	MHJ25022702	PV579839

In order to explore the phylogenetic position of the putative new species in *Ranunculus*, molecular analysis was performed based on 53 accessions (including 6 newly sequenced) from tribe Ranunculeae, tribe Anemoneae, tribe Helleboreae, tribe Callianthemeae, tribe Adonideae (Liu et al. 2018; Hu et al. 2024), and two species from Tribe Coptideae were chosen as outgroups, based on the results of Zhai et al. (2019). The complete plastome sequences were aligned using MAFFT v.7.402 (Katoh and Standley 2014) and then adjusted manually. Phylogenetic relationships were inferred using Bayesian Inference (BI) analyses and maximum likelihood (ML) based on complete plastome sequences. The ML phylogenetic tree was constructed using IQ‐Tree v.2.0.3 (Nguyen et al. 2015) by executing 5000 replicates of the SH approximate likelihood ratio test (SH‐aLRT) and ultrafast bootstrap (UFBS) (Hoang et al. 2018). The BI tree was conducted by MrBayes version 3.2.7 (Ronquist and Huelsenbeck 2003) using the settings: Bayesian trees were started from random trees; four Markov Chain Monte Carlo (MCMC) simulations were run simultaneously and sampled every 1000 generations for a total of 2 million. Runs were considered to have converged to stationarity when their average standard deviation of split frequencies was < 0.01 and the first 25% of trees were discarded as burn‐in. Finally, the tree files were visualized by the online tool of Interactive Tree Of Life (iTOL) (https://itol.embl.de/) v7 (Letunic and Bork 2021).

## 3 Results and Discussion

### 3.1 Morphology

*Ranunculus yuexiensis* is morphologically similar to *R*. *ternatus*. Although both species possess root tubers, *R*. *yuexiensis* can be readily distinguished from *R*. *ternatus* by several key characteristics: its carpels and achenes are puberulent (vs. glabrous), it has fewer basal leaves (2–5 vs. 5–10), larger flowers, longer petioles on the lower cauline leaves (vs. subsessile or sessile), and other distinguishing traits (Table 1). Morphological comparisons of *R*. *yuexiensis*, with the similar taxa *R*. *ternatus*, are provided in Table 2.

**TABLE 2:** Morphological comparisons between *R*. *yuexiensis* and *R*. *ternatus*.

Characters	*R. yuexiensis*	*R*. *ternatus*
Stem	12–25 cm tall	5–25 cm tall
Basal leaves	Basal leaves 2–5, 1.8–5.5 cm long, 2.2–6 cm broad, petioles 5–16 cm; blades simple, often 3‐lobulate	Basal leaves 5–10, 1.2–4.5 cm long, 2.4–6.5 cm broad, petioles 2–10 cm; blades simple or ternate
Cauline leaves	Lower cauline leaves with long petioles, upper cauline leaves subsessile or sessile	Sessile or subsessile
Flowers	Terminal, 1.6–2.4 cm in diameter	Terminal, 1–1.5 cm in diameter
Receptacles	Puberulent	Glabrous
Sepals	5–7 × 2.5–4.5 mm	3–4 × 1.2–3 mm
Petals	Elliptic to narrowly obovate, 8–12 × 5–7.5 mm	Narrowly obovate to obovate 5–7 × 3.5–4 mm
Petal nectary	Nectary pocket‐like with a scale	Nectary cup‐shaped without a scale or pocket‐like with an adaxial flap‐like scale
Carpels	Puberulent	Glabrous
Aggregate fruit	Subglobose	Subglobose
Achenes	Puberulent	Glabrous

### 3.2 Characteristics of Plastomes and Phylogenetic Analysis

**FIGURE 1:**
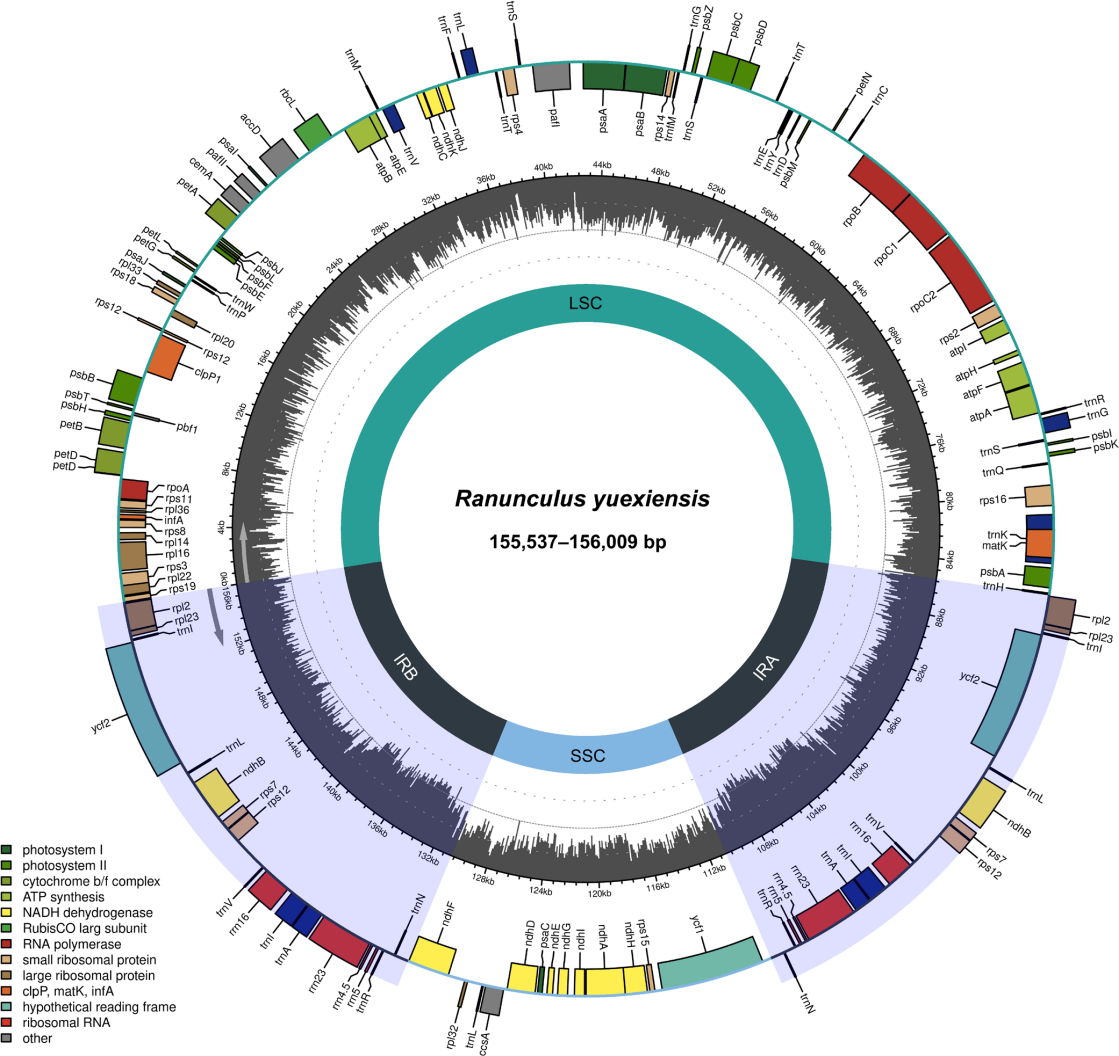
Plastome map of *Ranunculus yuexiensis*. Genes belonging to different functional groups are color‐coded. IR, inverted repeat; LSC, large single copy; SSC, small single copy.

The plastomes of *Ranunculus yuexiensis* samples ranged in length from 155,537 to 156,009 bp (GenBank: PV573921, PV573920, PV546306, Figure 1) and exhibited a typical quadripartite structure, consisting of the large single‐copy (LSC), small single‐copy (SSC), and two inverted repeat (IRa and IRb) regions. The plastome statistics across different populations of this new species are summarized in Table 3.

**TABLE 3:** Characteristics of plastomes of *Ranunculus yuexiensis*.

Characteristic	*Ranunculus yuexiensis*		
	RA1	RA2	RA3
Total length (bp)	156,009	155,537	156,001
GC%	38	38	38
LSC length (bp)	85,417	84,991	85,404
SSC length (bp)	19,794	19,784	19,799
IR length (bp)	25,399	25,381	25,399
Total genes	88	88	88
Protein‐coding genes	43	43	43
rRNA genes	8	8	8
tRNA genes	37	37	37

**FIGURE 2:**
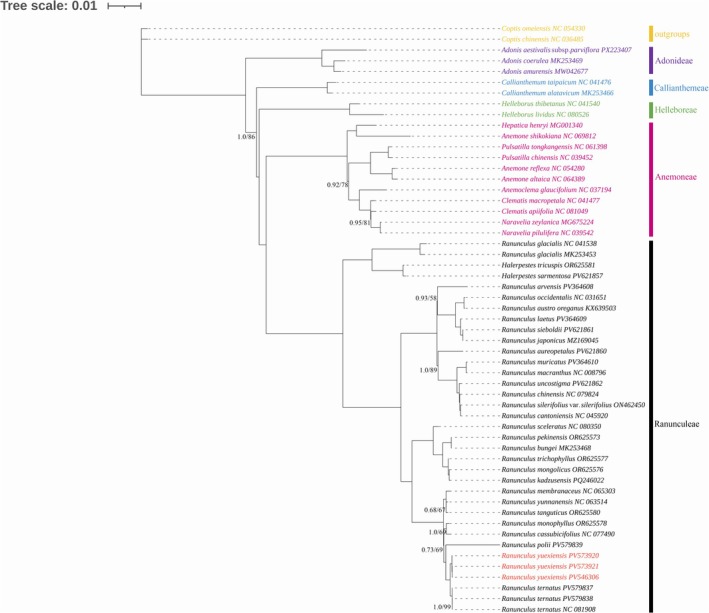
Phylogenetic trees constructed with the complete chloroplast genomes of 53 accessions from tribe Ranunculeae, tribe Anemoneae, tribe Helleboreae, tribe Callianthemeae, tribe Adonideae using the maximum likelihood (ML) and Bayesian inference (BI) methods. Two species from Tribe Coptideae were used as outgroups. Different colors are used to distinguish the tribes. Support values are indicated by the numbers above the nodes, with bootstrap support and posterior probability values shown for nodes with < 100% and 1.0 support, respectively. The new species is highlighted in red.

In recent years, phylogenies based on plastome data have served as key evidence for the delimitation of new species (Chen et al. 2024; Jiang et al. 2024; Wang et al. 2024). Based on complete chloroplast genomes, ML and BI trees were reconstructed and their topologies are similar (Figure 2). Both trees inferred from plastome data strongly supports the putative new species as a distinct lineage: all three accessions formed a monophyletic clade (MLBS = 100%, BIPP = 1) as sister to a well‐supported clade comprising three *R. ternatus* samples from geographically distinct populations (MLBS = 100%, BIPP = 1) (Figure 2).

## 4 Taxonomic Treatment

**FIGURE 3:**
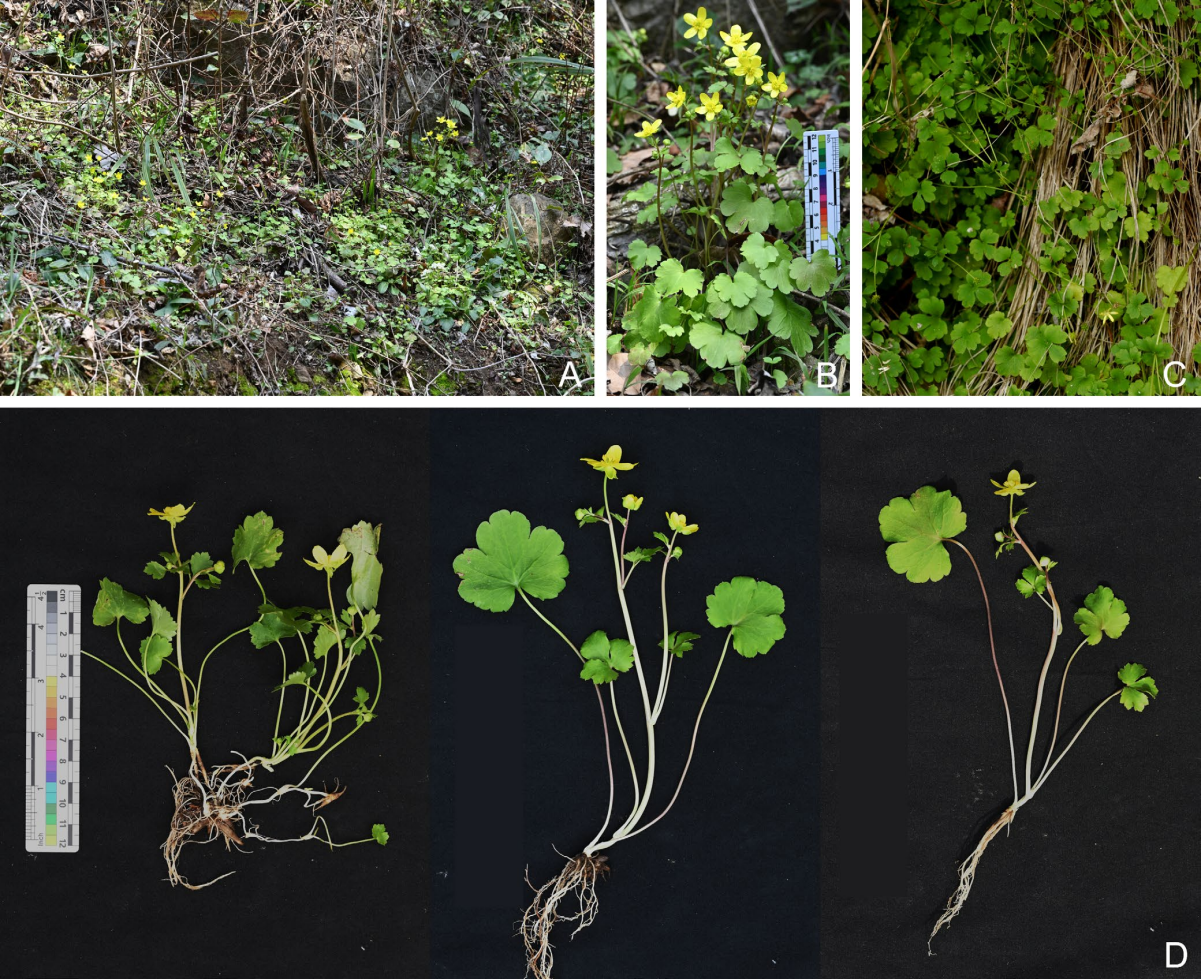
*Ranunculus yuexiensis* sp. nov. in the wild (A–C) habitat, (D) habit.

**FIGURE 4:**
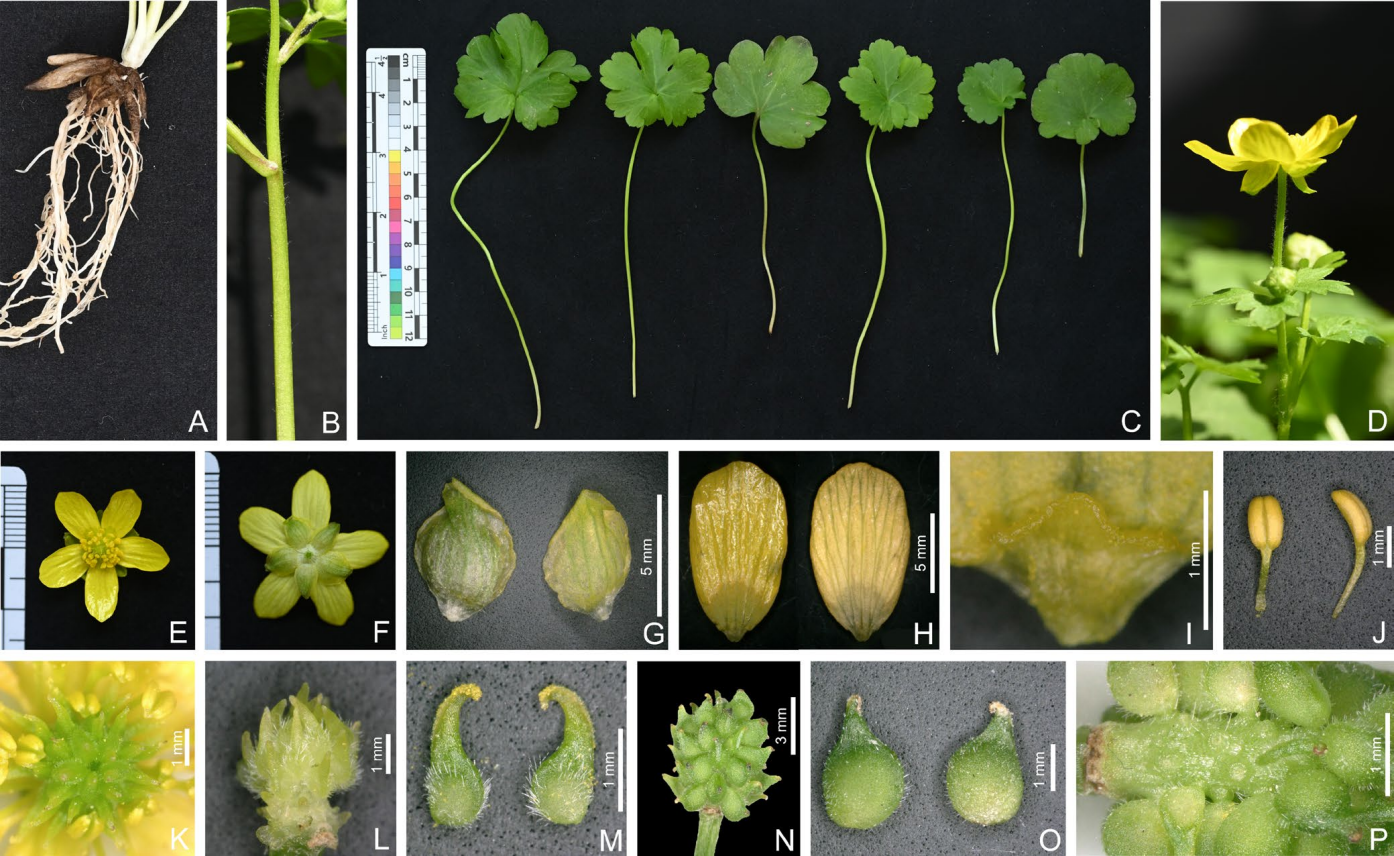
*Ranunculus yuexiensis* sp. nov. in the wild (A) roots, (B) portion of stem, (C) basal leaves, (D) flower (lateral view), (E) flower (top view), (F) flower (bottom view), (G) sepal (left: abaxial side; right: adaxial side), (H) petal (left: adaxial side; right: abaxial side), (I) petal nectary, (J) stamens, (K) gynoecia, (L) aggregate carpels, (M) carpels, (N) aggregate fruit, (O) achenes, (P) receptacle.

**FIGURE 5:**
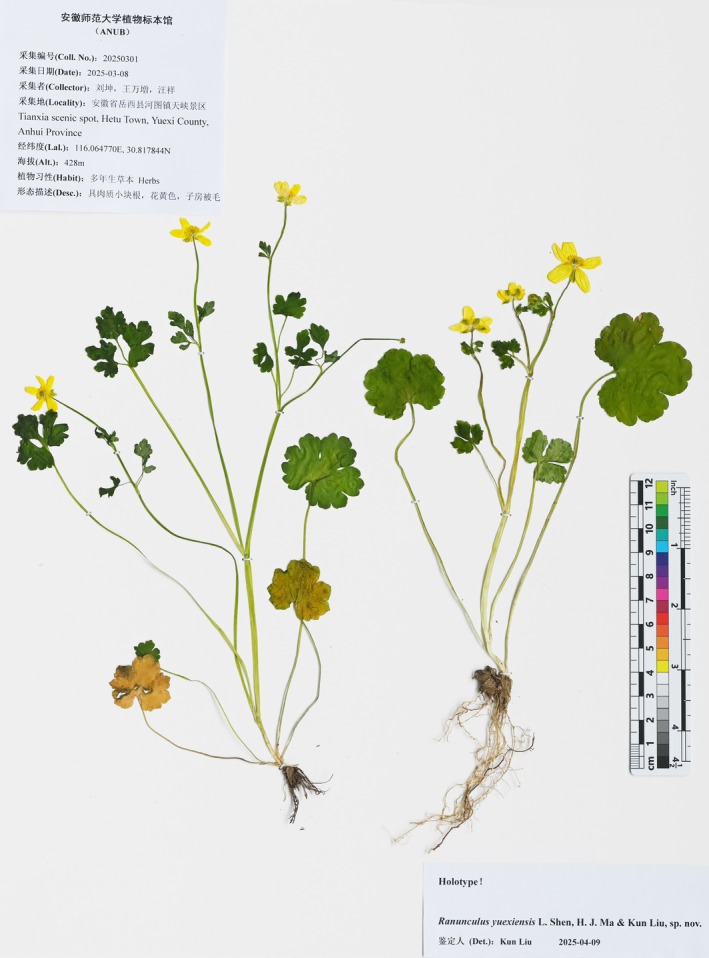
Photograph of the holotype of *Ranunculus yuexiensis* sp. nov.

*Ranunculus yuexiensis* L. Shen, H. J. Ma and Kun Liu, sp. nov. (Figures 3, 4, 5).

### 4.1 Type

CHINA. Anhui Province: Anqing City, Yuexi County, Hetu Town, Tianxia scenic spot, in moist places along streams and under broad‐leaved forests in valleys, 30.817844° N, 116.064770° E, 428 m alt., 8 March 2025, Liu20250301 (fl., holotype ANUB!, isotype ANUB! NAS! PE! KUN!).

### 4.2 Diagnosis

**FIGURE 6:**
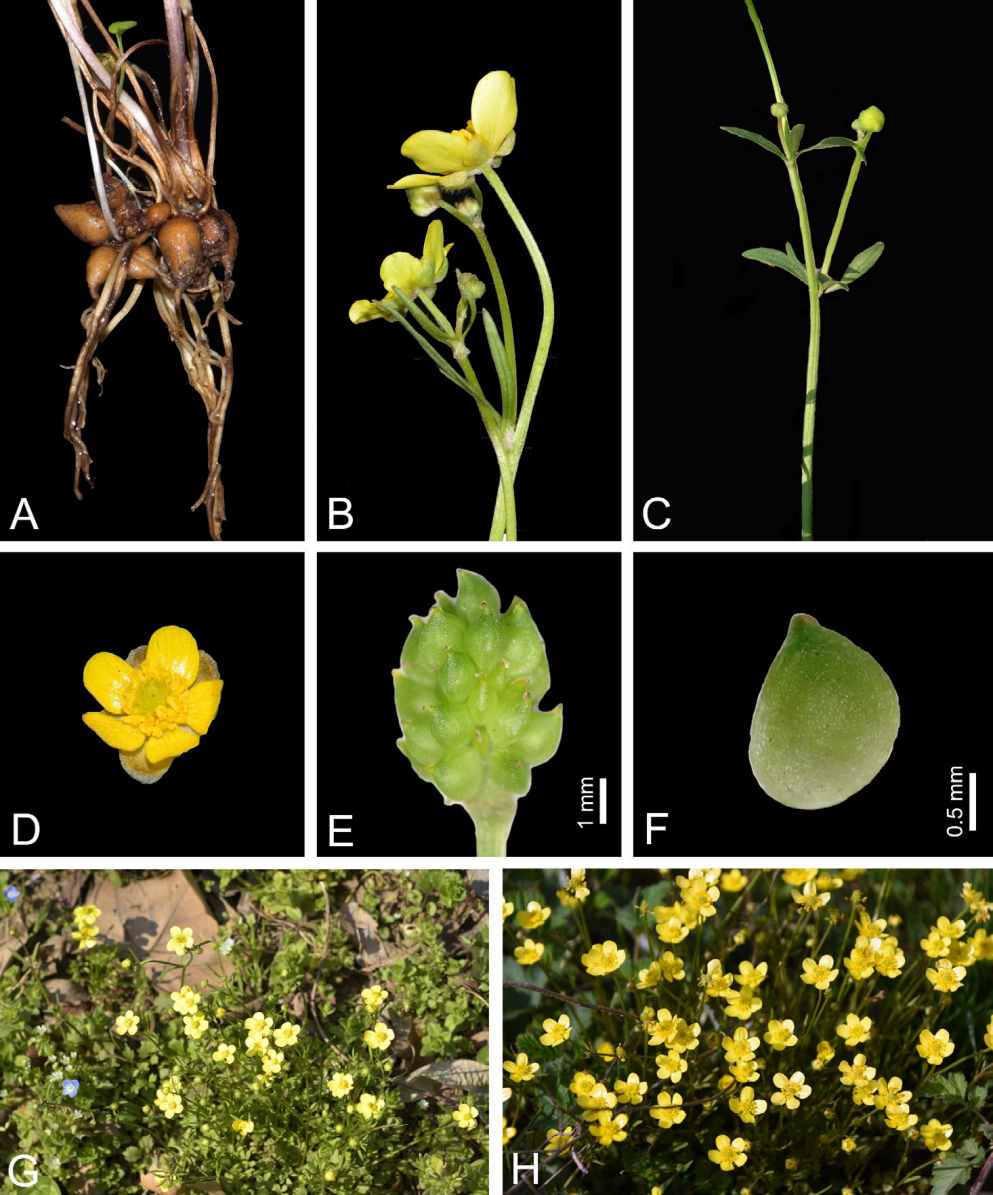
*Ranunculus ternatus* (A) roots, (B) flowers (side view), (C) cauline leaves, (D) flower (top view), (E) aggregate fruit, (F) achenes, (G) habitat, (H) plants.

*Ranunculus yuexiensis* (Figures 3 and 4) resembles *R*. *ternatus* (Figure 6), but differs by its simple and fewer basal leaves, long petioles on lower cauline leaves (vs. subsessile or sessile), larger flower size (1.6–2.4 cm vs. 1–1.5 cm in diameter), puberulent carpels and achenes (vs. glabrous) (Table 2).

**FIGURE 7:**
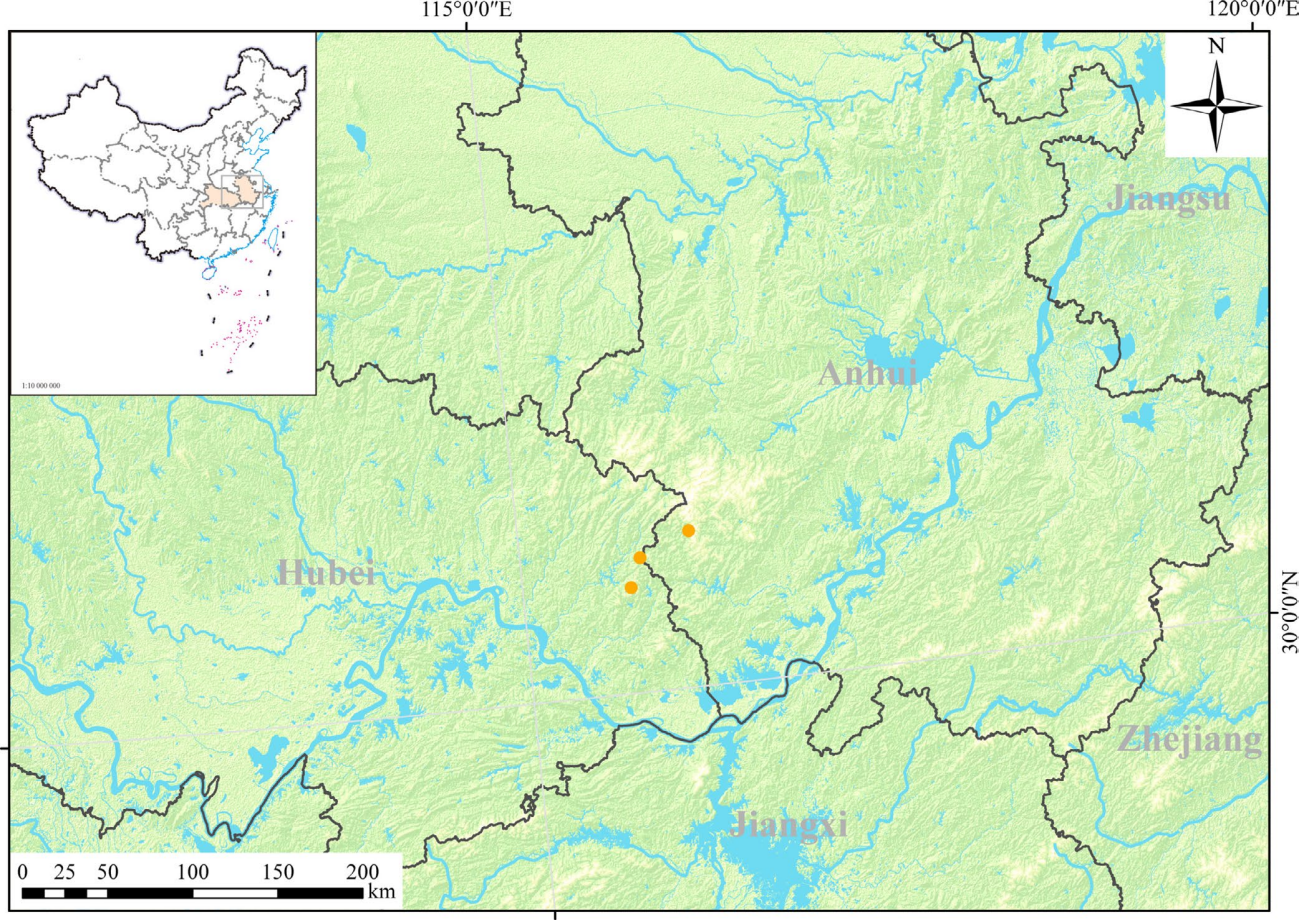
Distribution map of *Ranunculus yuexiensis* (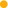).

### 4.3 Description

Herbs perennial. Roots tuberous and fibrous. Stems 12–25 cm tall, glabrous or sparsely puberulous, branched. Basal leaves 2–5; petioles 5–16 cm, glabrous or sparsely puberulous; blades simple, often 3‐lobulate, 1.8–5.5 cm long, 2.2–6 cm broad, subreniform or subpentagonal. Stem leaves 3–5, 3‐foliolate, adaxially glabrous or sparsely puberulous, abaxially glabrous, lower leaves long petiolate; upper leaves short petiolate or sessile. Flowers solitary, terminal, 1.6–2.4 cm in diameter. Receptacle puberulent. Sepals 5, rarely 6–7, yellowish‐greenish, elliptic, 5–7 mm long, 2.5–4.5 mm broad, abaxially glabrous to sparsely puberulent, reflexed or patent, concave. Petals 5, rarely 6–7, yellow, elliptic to narrowly obovate, 8–12 mm long, 5–7 (8) mm broad, at apex rounded, claw inconspicuous; nectary pit pocket‐like with a scale. Stamens numerous, 2–4 mm long; filaments linear, 1.8–2.5 mm long; anthers oblong, 1–2 mm long. Gynoecium ovoid, 2–4 mm long, 2–3 mm broad. Carpels 1–1.5 mm long; ovaries obliquely ovate to broadly ovate, 0.6–1 mm long, puberulent; styles 0.2–0.8 mm long, straight or slightly curved at apex. Aggregate fruit ovoid, 4–6 mm long, 4–5 mm broad. Achenes puberulent, ellipsoid‐orbicular to obovoid‐orbicular, 1.4–1.8 mm broad, 2–2.5 mm long, with styles beaked, 0.2–0.8 mm long.

### 4.4 Phenology

Flowering from early March and early April. Fruiting from late March and early May.

### 4.5 Etymology

The epithet “yuexiensis” is derived from the type locality, Yuexi County, Anhui Province, China. The Chinese name given is Yue Xi Mao Gen (岳西毛茛).

### 4.6 Distribution and Habitat

*Ranunculus yuexiensis* is currently known from its type locality, that is, Tianxia scenic spot in Yuexi county, Anhui province, China, and from the closely adjacent Yingshan county in Hubei province, China (Figure 7). It grows under broad‐leaved forests in valleys or on rocks in moist places along streams at altitudes of 250–450 m.

### 4.7 Preliminary Conservation Status

Our observations on the wild revealed that *Ranunculus yuexiensis* is very common in its type locality and adjacent areas. The species should better be considered as “Least Concern (LC)”, according to the IUCN Standards and Petitions Committee (IUCN 2022).

## Author Contributions

**Lang Shen:** data curation (equal), formal analysis (equal), investigation (equal), software (equal), visualization (equal), writing – original draft (equal). **Hai‐Jun Ma:** data curation (equal), investigation (equal), resources (equal), writing – original draft (equal). **Wei‐Qi Meng:** formal analysis (equal), methodology (equal), writing – original draft (equal). **Lu‐Jing Wang:** data curation (equal), investigation (equal), resources (equal). **Rong‐Bin Wang:** data curation (equal), investigation (equal), resources (equal). **Xiang Wang:** data curation (equal), investigation (equal). **Wan‐Zeng Wang:** data curation (equal), investigation (equal). **Guo‐Sheng Lv:** data curation (equal), methodology (equal), resources (equal). **Jian‐Wen Shao:** conceptualization (equal), methodology (equal), project administration (equal), supervision (equal), writing – review and editing (equal). **Kun Liu:** conceptualization (equal), formal analysis (equal), investigation (equal), methodology (equal), project administration (equal), supervision (equal), writing – original draft (equal), writing – review and editing (equal).

## Ethics Statement

The authors have nothing to report.

## Conflicts of Interest

The authors declare no conflicts of interest.

## Data Availability

The sequences of this study have been deposited in the National Center for Biotechnology Information (NCBI) database. GenBank accession numbers are provided in Table 1.
